# Carbon Nanotubes in TiO_2_ Nanofiber Photoelectrodes for High‐Performance Perovskite Solar Cells

**DOI:** 10.1002/advs.201600504

**Published:** 2017-01-20

**Authors:** Munkhbayar Batmunkh, Thomas J. Macdonald, Cameron J. Shearer, Munkhjargal Bat‐Erdene, Yun Wang, Mark J. Biggs, Ivan P. Parkin, Thomas Nann, Joseph G. Shapter

**Affiliations:** ^1^ School of Chemical EngineeringThe University of AdelaideAdelaideSouth Australia5005Australia; ^2^ School of Chemical and Physical SciencesFlinders UniversityBedford Park, AdelaideSouth Australia5042Australia; ^3^ Department of ChemistryUniversity College LondonWC1H OAJLondonUK; ^4^ Centre for Clean Environment and EnergyGriffith School of EnvironmentGold Coast CampusGriffith UniversityQueensland4222Australia; ^5^ School of ScienceLoughborough UniversityLoughboroughLECLE11 3TUUK; ^6^ MacDiarmid Institute for Advanced Materials and NanotechnologySchool of Chemical and Physical SciencesVictoria University of Wellington6140WellingtonNew Zealand

**Keywords:** carbon nanotubes, perovskite solar cells, photoelectrodes, photovoltaic, TiO_2_ nanofibers

## Abstract

1D semiconducting oxides are unique structures that have been widely used for photovoltaic (PV) devices due to their capability to provide a direct pathway for charge transport. In addition, carbon nanotubes (CNTs) have played multifunctional roles in a range of PV cells because of their fascinating properties. Herein, the influence of CNTs on the PV performance of 1D titanium dioxide nanofiber (TiO_2_ NF) photoelectrode perovskite solar cells (PSCs) is systematically explored. Among the different types of CNTs, single‐walled CNTs (SWCNTs) incorporated in the TiO_2_ NF photoelectrode PSCs show a significant enhancement (≈40%) in the power conversion efficiency (PCE) as compared to control cells. SWCNTs incorporated in TiO_2_ NFs provide a fast electron transfer within the photoelectrode, resulting in an increase in the short‐circuit current (*J*_sc_) value. On the basis of our theoretical calculations, the improved open‐circuit voltage (*V*_oc_) of the cells can be attributed to a shift in energy level of the photoelectrodes after the introduction of SWCNTs. Furthermore, it is found that the incorporation of SWCNTs into TiO_2_ NFs reduces the hysteresis effect and improves the stability of the PSC devices. In this study, the best performing PSC device constructed with SWCNT structures achieves a PCE of 14.03%.

## 1 Introduction

High‐performance photovoltaic (PV) cells that can convert the sun's energy directly into electricity through the PV effect are promising clean and renewable energy technologies and have great potential to address current energy related issues.^1^ Organolead halide (CH_3_NH_3_PbX_3_, X = I, Cl, or Br) implemented solar cells (known as perovskite solar cells (PSCs)) have received significant attention from both scientific and industrial communities and have become one of the most popular topics in scientific research.^2^, ^3^ This increasing popularity of PSCs is due to the unprecedented rapid progress that has been made in their power conversion efficiencies (PCEs) over a short period of time.^4^, ^5^ Since 2009, the PCEs of PSCs have increased from 3.8% to 22.1%, making them the fastest advancing PV technology.^6^, ^7^, ^8^, ^9^, ^10^ Recent successful fabrication of flexible and large‐area PSC devices shows great promise for the commercialization of this cutting‐edge PV technology.^11^, ^12^, ^13^, ^14^, ^15^, ^16^

The most commonly explored PSC architecture consists of a transparent conducting oxide (TCO) coated glass substrate, a compact titanium dioxide (TiO_2_) layer, mesoporous nanocrystalline TiO_2_ layer, perovskite layer, hole transporting layer, and metal contact.^5^, ^17^, ^18^, ^19^, ^20^, ^21^ The working principle of this class of PSCs can be expressed as follows: upon illumination, the perovskite is excited, producing an electron–hole pair. Then the electrons are injected into the conduction band of the *n*‐type semiconducting oxide (generally TiO_2_), while the holes are transported to the *p*‐type hole transporting materials (HTMs). Finally, the electrons and holes are collected at conductive electrodes such as TCO‐based anodes and metal cathodes, respectively.^22^ Fast charge‐transfer processes in PSCs are of particular importance to maximize the device performance. The measured values for the injection times of electrons and holes in PSCs are 0.4 and 0.6 ns, respectively.^23^ However, these values are three orders of magnitude longer than the hot carrier cooling time (≈0.4 ps), which leads to carrier trapping and a significant loss of the photon energy due to thermalization.^23^ Moreover, a large number of grain boundaries in the nanocrystalline films leads to rapid charge recombination, resulting in reduced device performance. These issues have led to some recent efforts focused on developing strategies to enhance the charge transport properties in PSCs.

One promising strategy is to use a 1D nanostructure as a substitute for the nanoparticles in the photoelectrode to suppress the charge recombination and provide a direct pathway along the long axis of 1D nanostructures for electron transport.^24^, ^25^, ^26^, ^27^, ^28^ In addition, the electron transport rate in 1D nanostructures such as nanofibers, nanowires, nanocolumns, and nanorods has been considered to be several orders of magnitude faster than that of nanoparticles.^29^, ^30^, ^31^, ^32^ This is achieved by reducing the scattering of free electrons from the grain boundaries of the interconnected nanoparticles.^33^ On the other hand, the incorporation of highly conductive carbon nanomaterials such as graphene and carbon nanotubes (CNTs) has also been proven to be an effective method to facilitate the charge transport and extend the electron lifetime, thereby enhancing the efficiency of PV devices.^22^, ^34^, ^35^, ^36^, ^37^ Although graphene and its derivatives have been successfully utilized for improving the performance of PSCs,^38^, ^39^, ^40^, ^41^, ^42^, ^43^ there has been no effort in the application of CNTs for use in PSC photoelectrodes. It should be noted that due to their unique structure and outstanding properties including excellent conductivity and high optical transparency, CNTs have exhibited promising results when they are used as an HTM and cathode in PSCs.^44^, ^45^, ^46^, ^47^, ^48^, ^49^ Moreover, CNTs are promising candidates for fabricating flexible fiber‐shaped PSCs.^50^, ^51^, ^52^, ^53^ Therefore, integrating highly conductive CNTs into 1D structured TiO_2_ for use in the photoelectrode of PSCs is an alternative approach to provide an ultrafast electron transport pathway to enhance device performance.

In the work presented here, the influence of CNTs on the performance of PSCs fabricated with 1D TiO_2_ nanofibers (NFs) is systematically examined. By using an optimal amount of single‐walled CNTs (SWCNTs) in the TiO_2_ NFs, a significant enhancement (≈40%) in the device performance is achieved as compared to the control cell fabricated without CNTs. Further, PCE enhancement is obtained by incorporating SWCNTs into both compact and mesoporous TiO_2_ layers of the PSCs. Based on our experimental and theoretical analysis, we attribute the performance enhancement of PSCs obtained by employing SWCNTs to the introduction of suitable energy levels and reduced charge recombination due to the increased charge transport of the photoelectrodes. More importantly, PSCs fabricated with SWCNT‐TiO_2_ NFs exhibited reduced hysteresis and improved stability both under light and during storage under humid conditions with respect to the control devices without SWCNTs. We also demonstrate in this study that SWCNTs can be used as an efficient HTM in PSCs. While previous work has shown TiO_2_ NF‐based PSCs can achieve PCE values of up to 9.8%^25^ and 13.4% for atomic layer deposited nanorods,^54^ in this work, our best performing PSC achieved a PCE of 14.03%.

## 2 Results and Discussion

**Figure 1:**
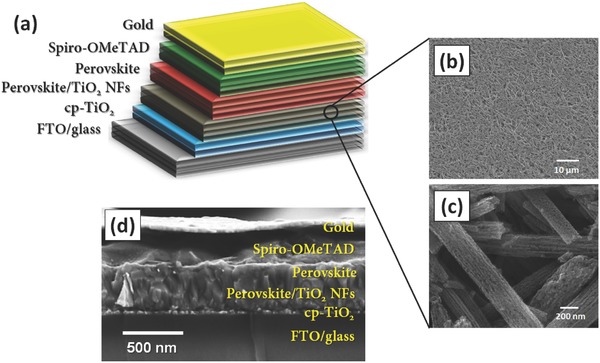
a) Schematic illustration of TiO_2_ NF photoelectrode based PSC. b) Low‐ and c) high‐resolution SEM image of TiO_2_ NFs. d) Cross‐sectional SEM image of representative TiO_2_ NF (400 nm thickness) photoelectrode based PSC.

In order to fabricate PSCs with the device architecture displayed in **Figure** **1**a, TiO_2_ NFs were first prepared using an electrospinning method. CH_3_NH_3_PbI_3_ was used as a light absorbing perovskite material. A detailed description of the synthesis process can be found in the Experimental Section. Low‐ and high‐resolution scanning electron microscopy (SEM) images of the prepared TiO_2_ NFs are shown in Figure 1b,c, respectively. The TiO_2_ NFs were several micrometres in length, while their diameter varied within a few hundreds of nanometres. In addition to the relatively uniform morphology, the prepared TiO_2_ NF films showed an excellent porous network, which can be beneficial for perovskite absorber loading (see Figure S1a–d, Supporting Information). The anatase phase of the TiO_2_ NFs on fluorine‐doped tin oxide (FTO) was confirmed by X‐ray diffraction as compared to the reference values (9853‐ICSD)^55^ and can be seen in Figure S1e (Supporting Information). In our devices (Figure 1d), TiO_2_ NFs were used as electron transporting layer (ETL).

The thickness of ETLs in the PSCs has a critical influence on the device performance.^10^, ^56^ To investigate the effect of the thickness of TiO_2_ NF films on the cell efficiency, five PSC devices were fabricated based on the TiO_2_ NF photoelectrodes with different thicknesses. The thickness of the TiO_2_ NF layer was controlled by dilution of the TiO_2_ NF paste. The diluted TiO_2_ solutions were spin‐coated onto the compact TiO_2_ (cp‐TiO_2_) with identical conditions. Cross‐sectional SEM images of TiO_2_ NF films with different thicknesses (≈285 to ≈2200 nm) are depicted in Figure S2a–e (Supporting Information). For comparison, a planar PSC device was fabricated without the TiO_2_ NF layer (Figure S2f and Figure S3, Supporting Information).

**Figure 2:**
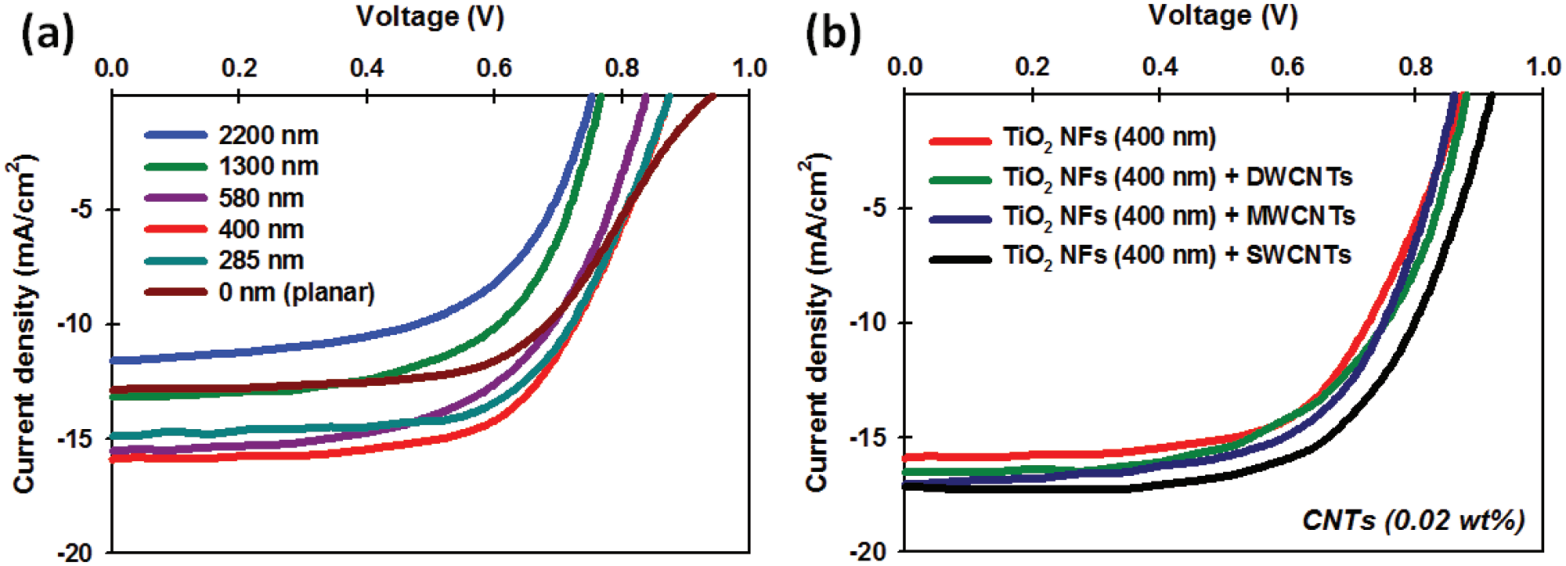
*J–V* curves of PSCs fabricated based on a) different thicknesses of TiO_2_ NF films and b) various types of CNT‐incorporated TiO_2_ NF photoelectrodes. For the fabrication of TiO_2_ NF–CNT photoelectrodes ≈400 nm TiO_2_ NFs was chosen.

The PV characteristics of the fabricated devices were studied under an air mass (AM) 1.5 illumination at 100 mW cm^−2^. The photocurrent density–voltage (*J–V*) characteristics of the PSCs fabricated with different TiO_2_ NF thicknesses are displayed in **Figure** **2**a and the corresponding PV parameters such as short‐circuit current (*J*_sc_), open‐circuit voltage (*V*_oc_), fill factor (FF), and PCE have been summarized in Table S1 (Supporting Information). As an optimal thickness of TiO_2_ NFs, the PSCs fabricated with ≈400 nm TiO_2_ NFs exhibited the highest PCE (average efficiency of 8.21 ± 0.46%). The observed *J*_sc_, *V*_oc_, and FF values for the best device based on ≈400 nm TiO_2_ NF photoelectrode PSCs were 15.91 mA cm^−2^, 0.87 V, and 0.62, respectively, yielding a PCE of 8.56%.

As shown in Figure 2a and Table S1 (Supporting Information), both *J*_sc_ and *V*_oc_ values of PSCs continuously decreased with the TiO_2_ NF film thickness (from ≈400 to ≈2200 nm), resulting in lower cell efficiencies. Increasing the film thickness, which in turn lowers the PCE, is believed to be a result of the high charge recombination rate within the TiO_2_ NF based devices.^10^, ^25^, ^57^ In contrast, the devices fabricated without TiO_2_ NFs (called planar PSCs) or with thin TiO_2_ NF layers (≈285 nm) showed high *V*_oc_, but their PCEs were low due to the decreased *J*_sc_ and FF values. This higher *V*_oc_ value of planar devices can be associated with the lower probability of charge recombination as compared to the porous structured PSCs. These results are very consistent with recent studies on CH_3_NH_3_PbI_3_‐based planar PSC devices.^25^, ^58^, ^59^ Considering the PCEs of the devices, the TiO_2_ NF (≈400 nm) film was chosen for further investigations and device fabrication.

To understand the effect of CNT types on the performance of PSCs, three different types of CNTs, namely double‐walled CNTs (DWCNTs), multiwalled CNTs (MWCNTs), and single‐walled CNTs (SWCNTs) were incorporated into the TiO_2_ NF photoelectrode based PSCs under the same experimental conditions. The *J–V* curves of these devices are plotted in Figure 2b. The concentration of CNTs in the TiO_2_ NF–CNT hybrid was 0.02 wt%. The average PCEs of these PSCs were calculated based on five identical devices (see Table S2 in the Supporting Information). It can be seen from Figure 2b and Table S2 (Supporting Information) that the incorporation of CNTs, regardless of their type, increases the *J*_sc_ value compared to the TiO_2_ NF‐only photoelectrodes, which can be associated with the high conductivity of CNTs.^36^ In particular, the use of the SWCNTs in TiO_2_ NF photoelectrodes was shown to considerably enhance the PCE of PSCs by improving the *J*_sc_ and *V*_oc_ values despite the fact that a very small amount of SWCNTs was added and no optimization was undertaken at this point. Indeed, the addition of SWCNTs (0.02 wt%) into the TiO_2_ NF photoelectrodes of PSCs increased the PCE from 8.56% to 9.91%. We hypothesize that this PCE enhancement is due to the excellent conductivity and mixture of metallic and semiconducting behaviour of SWCNTs. Therefore, the SWCNTs were chosen for further optimization of the devices to maximize the cell performance.

**Figure 3:**
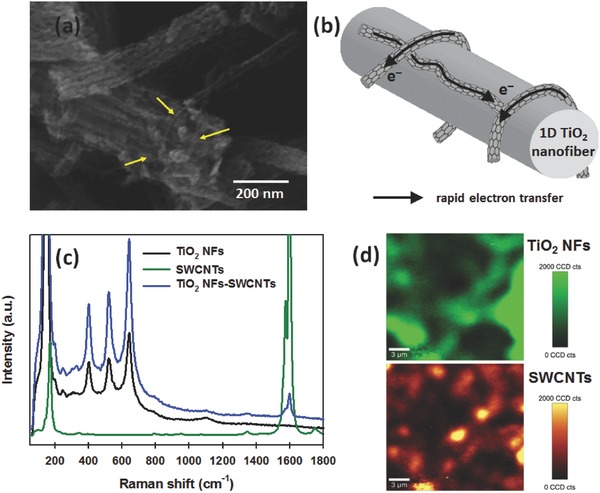
a) SEM image and b) schematic illustration of TiO_2_ NF‐SWCNT hybrid. c) Raman spectra of TiO_2_ NFs, SWCNTs, and TiO_2_ NFs–SWCNTs. d) Raman mapping image of TiO_2_ NFs (top, green) and SWCNTs (bottom, orange) in the hybrid sample. In both images, the “bright” regions represent the presence of the materials.

**Figure** **3**a displays the SEM image of SWCNT‐incorporated TiO_2_ NFs. SWCNTs a few nanometers in diameter (highlighted by yellow arrows in Figure 3a) are observed and wrapped around the TiO_2_ NFs, indicating successful integration of the SWCNTs into the TiO_2_ NFs. It is worth noting that 1D TiO_2_ NFs can provide a direct electron transport pathway.^24^ More importantly, in such hybrid structure (TiO_2_ NFs‐SWCNTs), the SWCNTs are expected to provide extremely fast electron transport pathway with excellent conductivity (see Figure 3b).

To further confirm the presence of SWCNTs in the hybrid, the samples were characterized using Raman microspectroscopy. Figure 3c shows the Raman spectra of TiO_2_ NFs, SWCNTs, and their hybrid structures. For the pure TiO_2_ NFs, four strong peaks at around 149, 397, 512, and 639 cm^−1^, which correspond to the E_g(1)_, B_1g(1)_, A_1g + B1g(2)_, and E_g(2)_ modes of anatase TiO_2_, respectively, were observed.^41^ On the other hand, the SWCNTs showed two typical Raman feature peaks at around 1346 and 1587 cm^−1^, which can be assigned to the “D” band (the disordered mode) and “G” band (tangential mode), in addition to the radial breathing mode peak at around 172 cm^−1^.^44^ For TiO_2_ NF‐SWCNT hybrid, all the Raman bands of both TiO_2_ NFs and SWCNTs were observed, further confirming the successful incorporation of SWCNTs into the TiO_2_ NF system. Moreover, confocal Raman spectral mapping was carried out on a selected area of the TiO_2_ NF‐SWCNT hybrid. Notably, the obtained maps in Figure 3d confirm the coexistence of TiO_2_ NFs and SWCNTs throughout the imaged area indicating a relatively homogeneous distribution of SWCNTs within the porous film.

**Figure 4:**
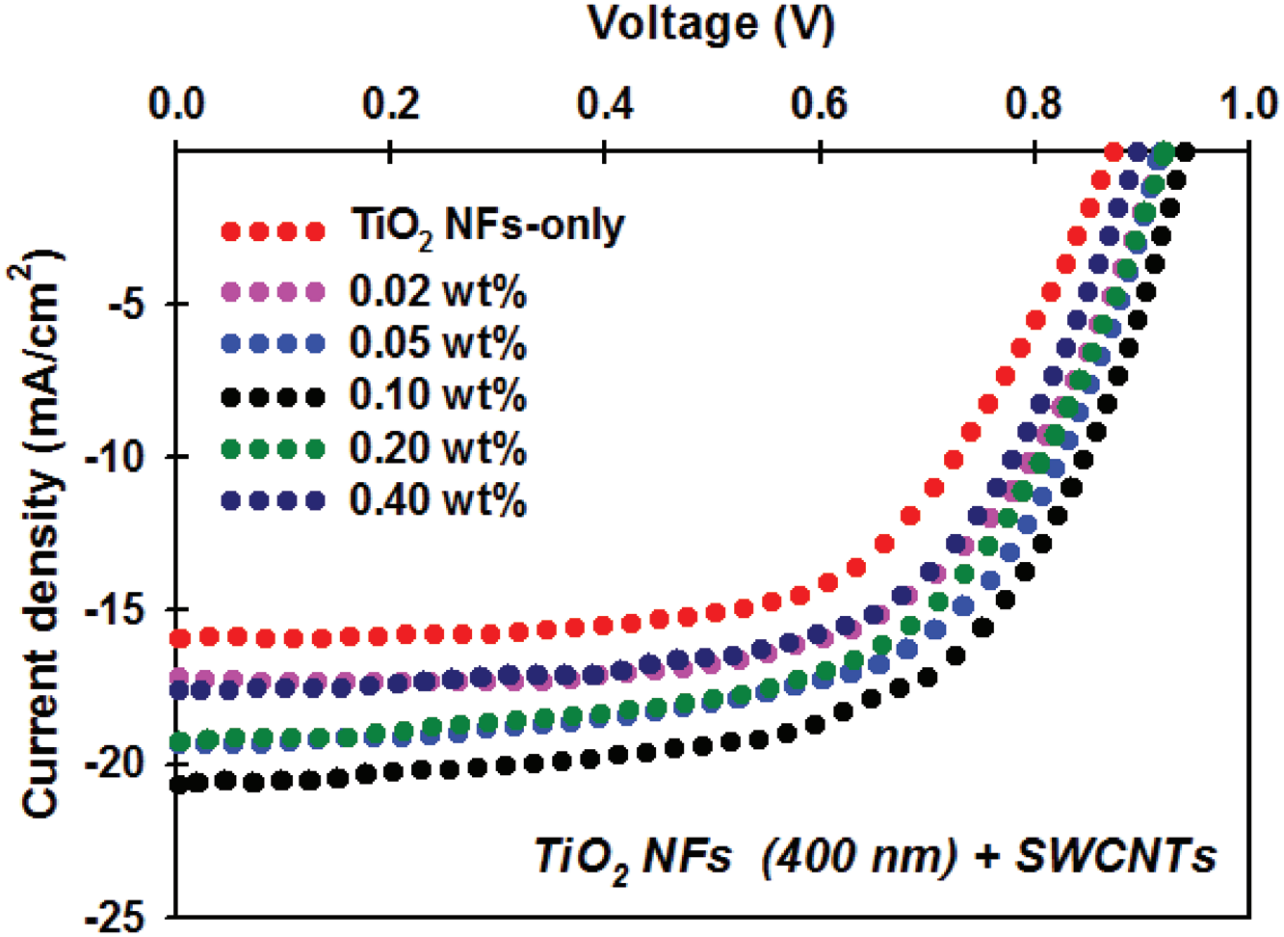
*J–V* curves of best performing PSC devices fabricated with different SWCNT content in the TiO_2_ NF photoelectrodes. The performance of the cells was measured under AM 1.5G illumination at 100 mW cm^−2^.

In order to optimize the devices, five PSCs were fabricated using different SWCNT content in the TiO_2_ NF photoelectrodes and their performances were compared with TiO_2_ NF‐only photoelectrodes control cells. **Figure** **4** shows the typical *J–V* curves of the TiO_2_ NF‐SWCNT hybrid photoelectrode based PSC devices. The corresponding PV parameters of these devices have been summarized in **Table** **1**. Starting from the control devices constructed based on TiO_2_ NF‐only photoelectrodes, the *J*_sc_ and *V*_oc_ are 15.91 mA cm^−2^ and 0.87 V, respectively. With increasing SWCNT loading in the TiO_2_ NF photoelectrodes, both the *J*_sc_ and *V*_oc_ values of the PSCs increased up to 20.68 mA cm^−2^ and 0.94 V, respectively, peaking at 0.10 wt%, followed by a decrease with further increases in SWCNTs content. We postulate that the increase in the *J*_sc_ of the cells is due to the improved conductivity of the films (see Figure S4 in the Supporting Information) that can accelerate the electron transport process within the photoelectrode of PSCs. However, when the concentration of SWCNTs in the hybrid further increases to 0.20 and 0.40 wt%, both *J*_sc_ and *V*_oc_ values of the devices decreased despite the films having reduced sheet resistance (*R*_s_).

**Table 1:** PV parameters of best performing PSC devices fabricated based on TiO_2_ NF photoelectrodes with different SWCNT loadings (extracted from the *J–V* characteristics reported in Figure 4). The average PCEs were calculated based on at least five devices. PV parameters of the best devices are highlighted in bold

Device	*J*_sc_ [mA cm^−2^]	*V*_oc_ [V]	FF	PCE [%]
TiO_2_ NF‐only	**15.91**;	**0.87**;	**0.62**;	**8.56**;
	15.44 ± 0.54	0.87 ± 0.01	0.61 ± 0.01	8.21 ± 0.46
0.02 wt% SWCNTs	**17.20**;	**0.93**;	**0.62**;	**9.91**;
	17.11 ± 0.20	0.92 ± 0.01	0.61 ± 0.01	9.69 ± 0.23
0.05 wt% SWCNTs	**19.34**;	**0.93**;	**0.61**;	**11.05**;
	19.04 ± 0.43	0.93 ± 0.00	0.61 ± 0.00	10.81 ± 0.34
0.10 wt% SWCNTs	**20.68**;	**0.94**;	**0.62**;	**12.03**;
	20.26 ± 0.37	0.94 ± 0.01	0.62 ± 0.01	11.51 ± 0.40
0.20 wt% SWCNTs	**19.24**;	**0.92**;	**0.60**;	**10.54**;
	19.16 ± 0.31	0.91 ± 0.01	0.59 ± 0.01	10.16 ± 0.32
0.40 wt% SWCNTs	**17.56**;	**0.90**;	**0.62**;	**9.80**;
	17.43 ± 0.37	0.89 ± 0.01	0.60 ± 0.02	9.40 ± 0.39

A series of detailed investigations have been carried out to understand the origin of the decrease in PV performance at higher SWCNT loadings. We measured the optical transmittance of the TiO_2_ NF films (on a glass slide) with different SWCNT loadings (Figure S5a, Supporting Information). A low optical transmittance of photoelectrode could elucidate the decreased *J*_sc_ value of the cells due to the less light being incident upon the perovskite layer. However, not surprisingly given the small amounts of CNTs being added, the changes in the film transmittance by adding SWCNTs into the TiO_2_ NFs were very small. For example, for the 0.20 wt% SWCNTs incorporated TiO_2_ NF film, the reduction of the transmittance was only ≈4% as compared to that of the TiO_2_ NF‐only film. Therefore, the decreased *J*_sc_ of the device after adding 0.20 and 0.40 wt% SWCNTs into the TiO_2_ NF photoelectrodes cannot be explained solely by the slightly reduced transmittance of the films. As with all other work that has incorporated nanocarbons in solar cells, we observed that there is an optimal loading which gives the maximum efficiency. At loading above the optimal, it is likely that the decreased PV performance is due to the fact that the CNTs provide extra junctions or sites where recombination of charge carriers is possible. It has been found that nanocarbons including CNTs are suitable candidates to replace the conventional HTMs in PSCs and/or improving the device efficiency owing to their fascinating properties.^44^, ^45^, ^46^, ^47^, ^48^, ^60^ In order to confirm our hypothesis, we used SWCNTs as an HTM for the TiO_2_ NF‐only photoelectrode based PSCs. For comparison, PSC devices with and without conventional HTM (Spiro‐OMeTAD) were fabricated and compared (Figure S5, Supporting Information). It is not surprising that the HTM‐free device (Perovskite/Au) exhibited a very poor PCE (3.65%), while Spiro‐OMeTAD (typical HTM) based PSC was able to achieve an average PCE of 8.21%. The poor performance of HTM‐free PSC is known to be due to the significant charge recombination caused by direct contact between the perovskite and gold electrode.^44^ Interestingly, when SWCNTs are used as an HTM in PSCs by inserting them between the perovskite and gold electrode, the fabricated device showed an improved PCE (6.01%) compared to the device without HTM, demonstrating that SWCNTs can act as an efficient HTL for PSCs. It is known that CNTs are ambipolar and can conduct both holes and electrons.^61^ This ability in PSCs is confirmed in these experiments. Thus, in the TiO_2_ NF‐SWCNT‐based electrode, it is very likely that the presence of larger amounts of SWCNTs will prolong the lifetime of the hole charge carriers and this will lead to increased recombination rates in the ETL. This likely explains the decreased *J*_sc_ and *V*_oc_ values of the PSC devices after adding high concentrations of SWCNTs into the TiO_2_ NF photoelectrodes.

As can be seen from Figure 4 and Table 1, the PSC devices fabricated based on the 0.10 wt% SWCNTs incorporated TiO_2_ NF photoelectrodes showed the highest PCE of 12.04% with an average efficiency of 11.51 ± 0.40%, whereas the control cells without SWCNTs displayed an average PCE of 8.21 ± 0.46%. The calculated PCE enhancement of PSCs loaded with 0.10 wt% SWCNTs (in comparison to the efficiency of the control cell) was 40.6%. In addition, the reproducibility of both control and SWCNTs incorporated devices was high and this is evident from the small standard deviation in the PV efficiency (Figure S6, Supporting Information). This result confirms that the incorporation of SWCNTs in the TiO_2_ NF photolectrodes does not alter the reproducibility of the devices. Indeed, the increased *J*_sc_ and *V*_oc_ values were the major contributions to this efficiency enhancement. Therefore, the devices loaded with 0.10 wt% SWCNTs and the control cells were chosen for further investigation to fully understand the role of SWCNTs in the PSCs.

**Figure 5:**
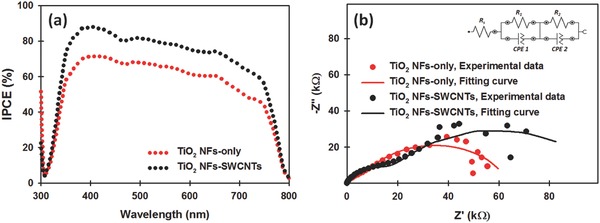
a) IPCE and b) EIS spectra of PSC fabricated with and without SWCNTs (0.10 wt%) in the TiO_2_ NF photoelectrode.

To confirm the enhancement of the *J*_sc_, the incident‐photon‐to‐current conversion efficiency (IPCE) spectra of the PSCs fabricated with and without SWCNTs (0.10 wt%) in the photoelectrodes were recorded and their results are plotted in **Figure** **5**a. Clearly, the IPCE value of TiO_2_ NF photoelectrodes containing SWCNTs is higher than that of the control cell without SWCNTs. The integrated photocurrent density of the TiO_2_ NF‐only and TiO_2_ NF‐SWCNT photoelectrode based PSCs was 15.20 and 19.50 mA cm^−2^, respectively, which are in agreement with the measured *J*_sc_ from the *J–V* characteristics of the devices. The improved current is evident over the entire wavelength region, indicative of enhanced electron collection in the PSC loaded with SWCNTs.

The improved *J*_sc_ value of the TiO_2_ NF‐SWCNT photoelectrodes was further examined using electrochemical impedance spectroscopy (EIS). EIS measurement of full PSC devices in the dark can be used to distinguish the charge transfer at the perovskite/HTM/cathode interface and the charge recombination at the ETL (TiO_2_)/perovskite interface.^62^ EIS of the PSCs fabricated with and without SWCNTs in the TiO_2_ NF photoelectrodes was measured at a bias of 0.3 V in the dark and the extracted data with a simplified circuit model are illustrated in Figure 5b. In general, the high frequency arc is associated with the diffusion of holes through the HTM, while the lower frequency arc is related to the recombination resistance, *R*_rec_, mainly due to the charge recombination between the electron transporting material and HTM.^62^, ^63^ Clearly, the diameter of the semicircle at the lower frequency of SWCNT‐incorporated TiO_2_ NF photoelectrode based PSC is larger than that of TiO_2_ NF‐only device, indicating higher *R*_rec_ (87.0 kΩ) for SWCNTs employed device as compared to the control device (58.3 kΩ). The high *R*_rec_ indicates an efficient blocking for possible recombination. This result clearly demonstrates that the SWCNTs significantly reduced the charge recombination and increased the charge transfer within the cell as expected.

To further investigate the effect of SWCNTs on the charge transfer process of TiO_2_ NF photoelectrode based PSCs, dark *J–V* measurements were carried out (see Figure S7 in the Supporting Information). The dark *J–V* measurement can provide important information about the recombination process in the devices.^42^ From the dark *J–V* measurement, an ideality factor of each device can be calculated, and notably, low values of ideality factor represent less charge recombination. The ideality factor of the TiO_2_ NF‐SWCNT photoelectrode based PSC device was 1.82, which was lower than that (2.41) of the control PSC, proving that the incorporation of SWCNTs in the TiO_2_ NFs significantly suppresses the recombination process in the devices.

**Figure 6:**
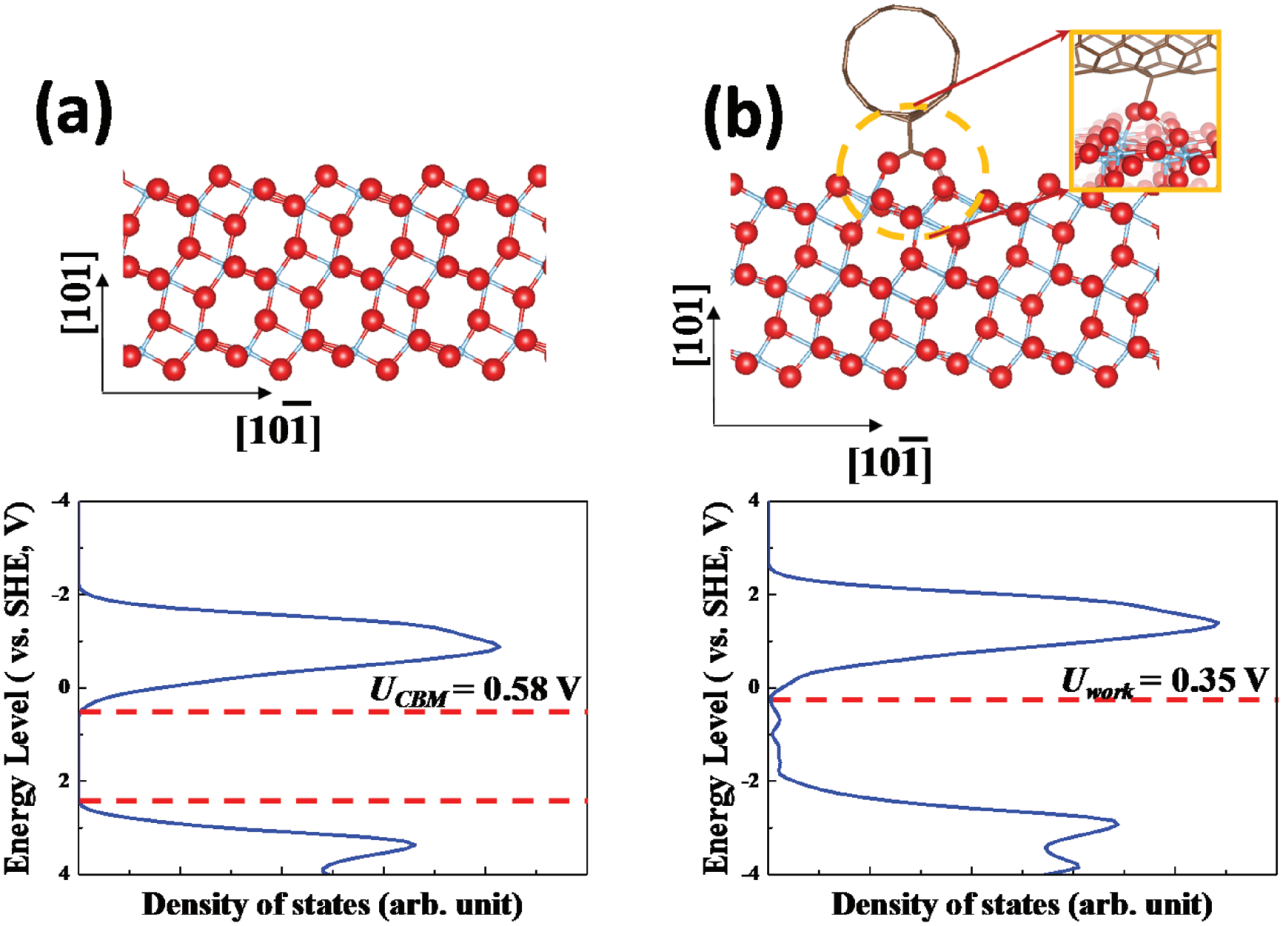
a) Atomic structure of pure anatase TiO_2_ (101) surface (upper panel) and its DOS plot versus the SHE in V (lower panel). b) Atomic structure of SWCNT–anatase TiO_2_ (101) surface (upper panel) and its DOS plot versus the SHE in V (lower panel). Inset in (b) is the atomic structure at the interface between the SWCNT and TiO_2_ (101). Blue, Ti; red, O; brown, C; pink, H.

It should be noted that the previous studies have shown decreased and/or unchanged *V*_oc_ values of PSC devices after the incorporation of various nanocarbon materials into the ETLs.^39^, ^40^, ^42^ Interestingly, our study demonstrates that the addition of a small amount of SWCNTs into the ETL leads to a considerable enhancement in the *V*_oc_. In order to explain why the *V*_oc_ value increased after adding SWCNTs into the TiO_2_ NFs, we investigated the interactions between SWCNTs and anatase TiO_2_ (101) surface using a computational method based on the first principles density functional theory (DFT). In theory, the *V*_oc_ of PSCs is the difference between the conduction band minimum (CBM) of the TiO_2_ and the potential energy of the HTM. Based on the DFT results, the CBM level is 0.58 V versus standard hydrogen electrode (SHE) (see **Figure** **6**a). After the adsorption of SWCNT, the analysis of density of states (DOS) demonstrates that the system changes from a semiconductor to a metallic material (see Figure 6b), which supports the improved electronic conductivity observed in EIS measurements. Moreover, the theoretical *V*_oc_ can be calculated based on the difference between the work function of metallic SWCNT‐TiO_2_ and the potential energy of the HTM. The theoretical results reveal that the work function of SWCNT‐TiO_2_ is 0.35 V versus SHE, which is 0.23 V higher than the CBM level of TiO_2_. Since the redox potential of the HTM is a constant, the theoretical *V*_oc_ can, therefore, be enhanced by 0.23 V, which matches the experimental observations.

**Figure 7:**
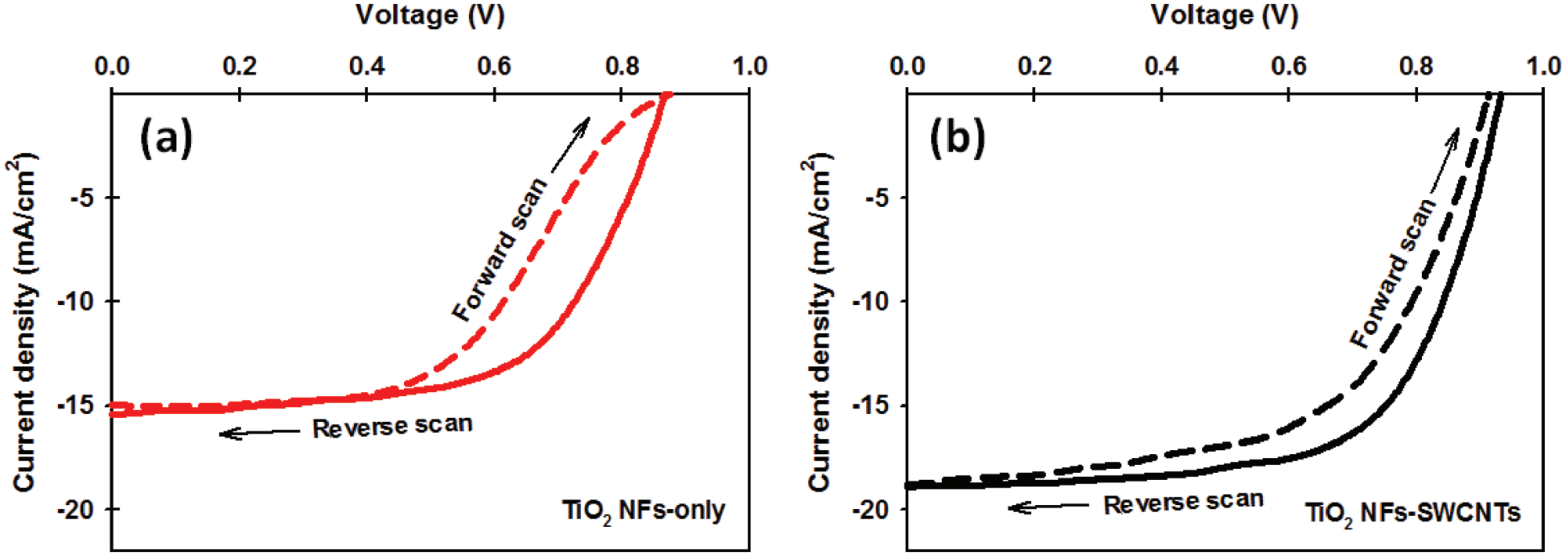
*J–V* curves measured with FS and RS for the a) TiO_2_ NF‐only and b) TiO_2_ NF‐SWCNT photoelectrode based PSCs. Detailed PV parameters are given in Table S3 (Supporting Information).

One of the critical challenges in PSCs is hysteretic *J−V* behaviour. In general, the hysteresis effect in PSCs is observed from the forward scan (FS, from *J*_sc_ to *V*_oc_) and reverse scan (RS, from *V*_oc_ to *J*_sc_) of *J–V* measurement.^64^ Here, we studied the hysteresis behaviour of our PSCs fabricated with and without SWCNTs in the TiO_2_ NF photoelectrodes (see **Figure** **7**). As illustrated in Figure 7a, the TiO_2_ NF‐only‐based device exhibited a large hysteresis and distortion in the *J–V* curves. Such hysteresis behaviour causes an underestimation of the real *J–V* curves in the FS and overestimation in the RS. Interestingly, it can be seen from Figure 7b and Table S3 (Supporting Information) that the incorporation of SWCNTs into the photoelectrodes reduces the hysteresis behaviour of the cells. The exact mechanism of hysteresis phenomenon in PSCs is not well established, however, several explanations have been suggested based on both experimental and theoretical investigations.^64^ It has been suggested that an anomalous hysteresis in PSCs can be attributed to the charge recombination at the interface between perovskite and charge transporting layer.^42^, ^65^ Clearly, SWCNTs suppressed the charge recombination in the PSC photoelectrodes and this may contribute to the reduced *J–V* curve hysteresis.

**Figure 8:**
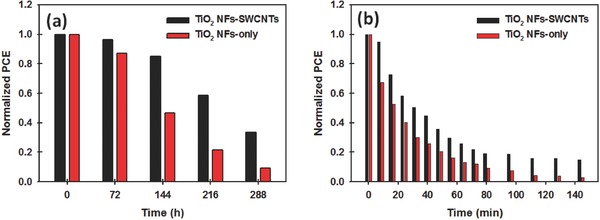
a) Normalized PCE of PSCs fabricated with and without SWCNTs in the TiO_2_ NF photoelectrodes as a function of long‐term storage time. The unencapsulated cells were kept in the dark in ambient conditions at a relative humidity of at least 60%. b) Normalized PCE of the devices with and without SWCNTs in the TiO_2_ NF photoelectrodes as a function of time exposed to continuous light illumination (100 mW cm^−2^, xenon lamp) for 144 min.

The stability of PSCs is an important factor for their potential commercialization on an industrial scale. The long‐term storage stability of the PSC devices fabricated with and without SWCNTs in the TiO_2_ NF photoelectrodes was investigated over 12 d (288 h). For the long‐term storage stability test, the unencapsulated cells were stored in the dark and kept in ambient conditions (normal laboratory) at a relative humidity of at least 60%. Normalized PCEs of these two devices are plotted in **Figure** **8**a and detailed PV parameters (*J*_sc_, *V*_oc_, FF, and PCE) are also shown in Figure S8a (Supporting Information). It can be seen from Figure 8a that the PCE of the control PSC without SWCNTs in the photoelectrode dropped by ≈90% after 288 h, while the TiO_2_ NF‐SWCNT photoelectrodes exhibited ≈66% degradation after the same period. Similar phenomena were also observed in several recent reports using graphene derivatives in the TiO_2_ photoelectrodes of PSCs.^42^, ^43^ It is now accepted that in a humid environment, water molecules cause the decomposition of perovskite and result in severe morphological changes (such as pinholes, small grains, and coarse surface).^66^ Such morphological features are detrimental to the direct electron transfer between perovskite and TiO_2_. The presence of SWCNTs in the TiO_2_ photoelectrodes provides better connectivity with the perovskite, and hence provides extra charge carrying pathways, which may mitigate the changes in the perovskite structure. Undoubtedly, this extends the electron lifetime in the cell helping maintain efficiency over longer times.

In addition to the long‐term storage stability in ambient conditions, the light stability of the PSCs with and without SWCNTs was explored. This was achieved by exposing PSCs to continuous light illumination (100 mW cm^−2^, xenon lamp) for 144 min. The data were collected in the reverse scan direction every 8 min in an ambient atmosphere. Detailed PV parameters are also shown in Figure S8b (Supporting Information). It can be clearly observed from Figure 8b that the device fabricated with TiO_2_ NF‐SWCNT photoelectrodes showed relatively better stability than the control cell based on TiO_2_ NF‐only photoelectrode. This improved light stability of the device with SWCNTs in the photoelectrode may be attributed to the high thermal conductivity of SWCNTs. The highly conductive SWCNTs are expected to effectively remove the heat during cell operation (during light soaking), which will likely help stability of the devices during operation.

**Figure 9:**
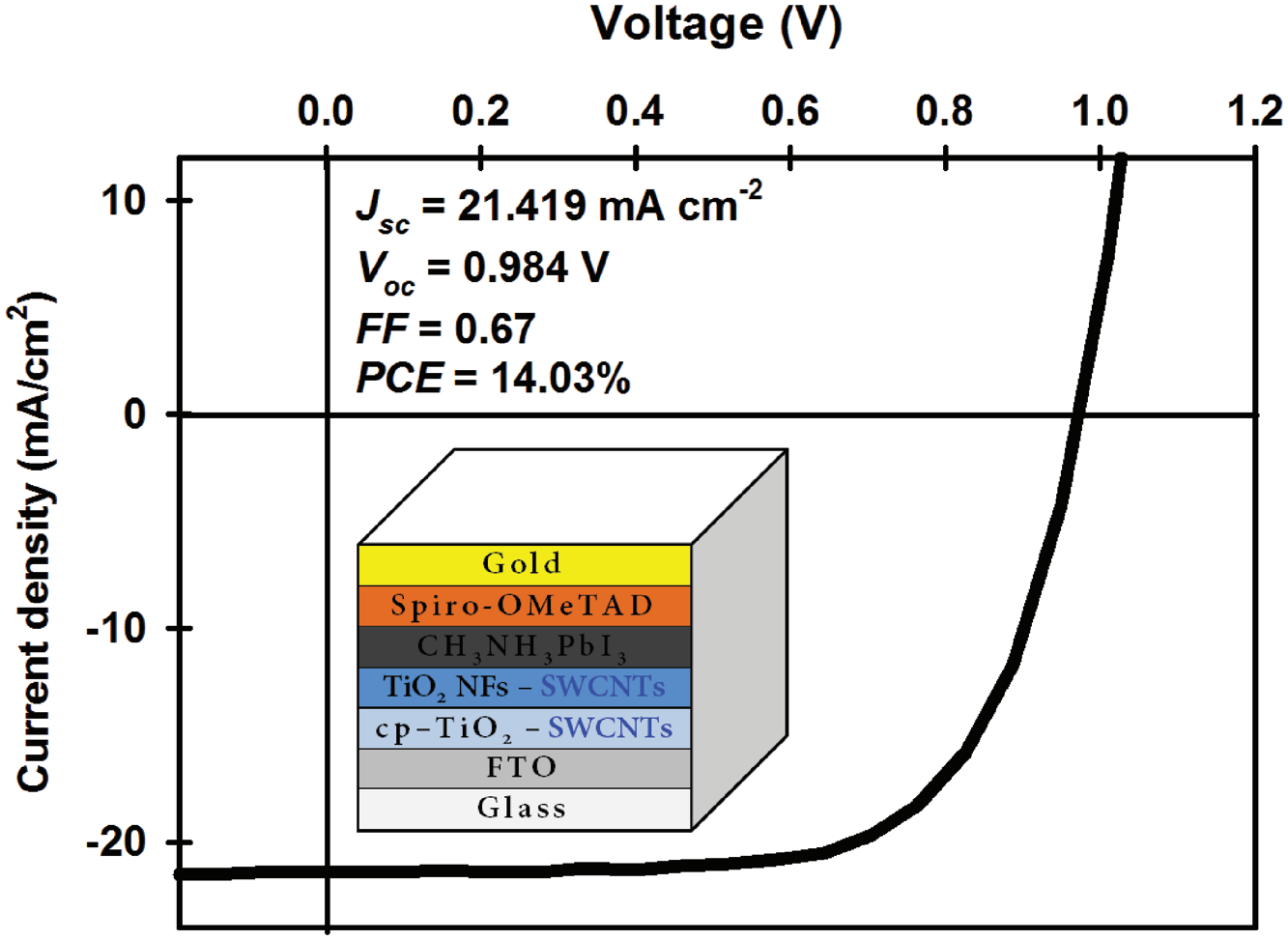
*J–V* curve of the best performing device in this study. Inset shows the device structure and detailed PV parameters.

It has been well established that the use of nanocarbons in the compact TiO_2_ (cp‐TiO_2_) layer of PSCs is an effective strategy to enhance the performance of PSC devices.^38^, ^40^ Therefore, in this work, we also explored the influence of SWCNTs in the cp‐TiO_2_ layer on the efficiency of TiO_2_ NF‐only photoelectrode based PSCs. As compared to the control device without any SWCNTs (Figure S9a, Supporting Information), SWCNT‐incorporated cp*‐*TiO_2_ layer based TiO_2_ NF photoelectrode based device exhibited a clear enhancement in the efficiency (see Figure S9b and Table S4 in the Supporting Information). We postulate that this improvement in the efficiency of PSCs is due to the enhanced electron transport rate and thermodynamically favourable energy transfer path within the photoelectrode.^40^ Furthermore, in order to maximize our device performance, we fabricated PSC devices with SWCNTs in both cp*‐*TiO_2_ layer and TiO_2_ NF layer. The layered structure and PV characteristics of the device are illustrated in **Figure** **9**. The observed *J*_sc_, *V*_oc_, and FF values for this device were 21.42 mA cm^−2^, 0.98 V, and 0.67, respectively, yielding a PCE of 14.03%. It should be pointed out that while this enhanced PCE is not over the 20% being reported for the best cells,^8^ the considerable improvement in PCE using CNTs in combination with a 1D nanomaterial does point to a promising research direction where other materials could be used for these systems and as such the improved PCE observed is an important result.

It should be noted that the *V*_oc_ values of our devices were slightly lower than those reported in other studies,^24^, ^25^ despite optimization. It was found that the aperture masking of the PSCs during the *J–V* analysis has some influence on the *V*_oc_ of the devices, which leads to an underestimation and/or overestimation of cell performance.^67^ We note that the aperture mask with an area of 0.081 cm^2^ was used for all above *J–V* measurements in this work to provide an accurate comparison of PSC devices, while the active area (overlapped area of FTO electrode and gold electrode) of the devices was 0.14 cm^2^. In order to understand the effect of the aperture mask on the PV parameters of our devices, the control device based on TiO_2_ NF‐only photoelectrodes and the best performing PSC (device structure shown in the inset of Figure 9) were fabricated and their *J–V* characteristics are analyzed with and without an aperture mask. It can be observed from Figure S10 (Supporting Information) that the *V*_oc_ values of PSCs were increased by ≈53 mV when measurement is carried out without an aperture mask, but no changes were observed in the *J*_sc_ and FF values. For example, the *V*_oc_ of the TiO_2_ NF‐only PSCs (control cell) increased from 0.87 to 0.93 V when *J–V* characteristics are measured without a mask, and yielding an improved PCE (9.1%). These improved values (*V*_oc_ and PCE) are comparable and similar to those reported in the literature,^24^, ^25^ suggesting that in our study, the *V*_oc_ values measured with aperture mask were slightly underestimated and would be higher if no mask was applied during the *J–V* analysis.

## 3 Conclusion

In this work, we have demonstrated the successful incorporation of highly conductive CNTs into 1D TiO_2_ NF photoelectrodes for highly efficient PSCs. We found that the use of SWCNTs is the most effective material among three different types of CNTs to obtain high PCEs from the devices. In comparison to the control device fabricated without CNTs, a significant enhancement (≈40%) in the cell efficiency is achieved by incorporating an optimized amount of SWCNTs into the TiO_2_ NF PSCs. The improved *J*_sc_ and *V*_oc_ values are the main contributions to this efficiency enhancement. The increased *J*_sc_ is due to the fact that hybrid structure of TiO_2_ NF‐SWCNT provided a fast electron transport pathway with excellent conductivity and thus suppressed the charge recombination rate in the PSCs. Our theoretical calculation revealed that the energy level of the photoelectrode is changed by introducing SWCNTs into the TiO_2_ NFs, and resulted in an increase in the *V*_oc_. Interestingly, we found that the use of SWCNTs in the TiO_2_ NF photoelectrode reduced the hysteresis behaviour and improved the stability of the PSC devices both during operation under light and for long‐term storage in humid conditions. Importantly, our best performing PSC device fabricated with SWCNTs in both cp‐TiO_2_ and TiO_2_ NF layers achieved a PCE of 14.03%. Therefore, we believe that this work will open new research avenues for the development of nanocarbon and nanofiber materials in PSCs.

## 4 Experimental Section

*Materials*: Unless otherwise stated, all chemicals and reagents were purchased from Sigma‐Aldrich. An FTO‐coated glass electrode, methylammonium iodide (CH_3_NH_3_I), and tris(1‐(pyridin‐2‐yl)‐1*H*‐pyrazol)cobalt(III)*tris*(hexafluorophosphate) (FK102 Co(III) PF6) salt were obtained from Dyesol. 2,2′,7,7′‐Tetrakis‐(*N,N*‐di‐*p*‐methoxyphenylamine)‐9,9′‐spirobifluorene (Spiro‐OMeTAD) was purchased from Solaronix. Arc‐discharge SWCNTs (P3‐SWNT) were purchased from Carbon Solution Inc., Riverside, CA, USA, while both DWCNTs (<5 nm in diameter) and MWCNTs (<10 nm in diameter) with a purity of 90% and a length of 5–15 µm were obtained from Shenzhen Nanotech Port Co., Ltd, China.

*Materials Preparation*: TiO_2_ NFs were prepared by electrospinning a sol–gel solution consisting of titanium(IV) *n*‐butoxide (TIB, 5.0 g), poly(vinyl pyrrolidone) (PVP, 1.0 g), and glacial acidic acid (1 mL) in an ethanol (10 mL). A detailed description of the TiO_2_ NF synthesis process can be found in the literature.^37^ The obtained TiO_2_ NFs were further used to prepare a viscous paste according to the established procedure.^37^ The concentration of the TiO_2_ NFs in the paste was calculated to be ≈15.14 wt%. To optimize the thickness of the TiO_2_ NF layer in the photoelectrodes, the prepared viscous paste was diluted with various amounts of ethanol for various times, followed by spin‐coating at 2500 rpm for 30 s. Dilution ratios of TiO_2_ NF paste and ethanol, namely 1:1, 1:2, 1:4, 1:6, and 1:8 (weight ratio), were used to prepare films with thicknesses of 2200, 1300, 580, 400, and 280 nm, respectively. On the basis of PV performance obtained using these diluted solutions, the dilution ratio of 1:6 (paste/ethanol) was chosen.

In order to prepare the stock solution of CNTs, an aqueous Triton X‐100 solution with 1 vol% concentration was first prepared. Then, CNTs (10 mg) were dispersed in the previously prepared Triton X‐100 solution (10 mL) using an ultrasonication (bath) for 1 h. The concentration of the CNT stock solution was 1 mg mL^−1^. For the preparation of TiO_2_ NF–CNT hybrid based solutions, the desired concentration (wt%) of CNTs in the hybrid was obtained by adding an appropriate volume of the CNT stock solution into 3.50 g of the diluted TiO_2_ NF dispersion.

*Device Fabrication*: First, FTO‐coated glass substrates were etched using 2 m HCl and Zn powder, followed by sequential cleaning with a detergent (Pyroneg) and washing with acetone, ethanol, and Milli‐Q water under ultrasonication for 10 min each. After drying the cleaned FTO electrodes with a stream of nitrogen gas, a thin compact layer of TiO_2_ was deposited onto the FTO substrate by spin‐coating 0.15 and 0.25 m titanium diisopropoxide bis(acetylacetonate) (75 wt% in isopropanol, Aldrich) in 1‐butanol solution. After each spin‐coating, the films were dried by heating at 150 °C for 15 min in air. For the fabrication of our best performing PSC (device structure is shown in the inset of Figure 9), SWCNTs were added into the titanium diisopropoxide bis(acetylacetonate) in 1‐butanol solution to prepare the precursor for the compact layer. The concentration of the SWCNTs in the composite was 0.02 wt%. Then, a thick ETL was deposited onto the compact layer to prepare the photoelectrode by spin‐coating a solution of TiO_2_ NF‐only and/or TiO_2_ NF‐CNT at 2500 rpm for 30 s. The films were heated gradually under an air flow at 125 °C for 5 min, 325 °C for 5 min, 375 °C for 15 min, and 450 °C for 1 h, followed by cooling to room temperature. The photoelectrode films were immersed in 40 × 10^–3^ m TiCl_4_ aqueous solution at 70 °C for 30 min and dried with nitrogen gas, which was again annealed at 450 °C for 1 h. After cooling to ≈120 °C, the films were transferred into a nitrogen‐filled glove box.

The perovskite precursor solution was prepared by dissolving a stoichiometric amount (1:1 molar ratio) of PbI_2_ (0.507 g) and CH_3_NH_3_I (0.175 g) in an anhydrous dimethylsulfoxide (DMSO, 1 mL). The perovskite layer was deposited onto the photoelectrode films by spin‐coating as described in the literature.^68^ The spin‐coating recipe includes two steps, first 1000 rpm for 10 s with a ramp of 250 rpm s^−1^, then 5000 rpm for 30 s with a ramp of 2000 rpm s^−1^. ≈12 s before the end of the spin‐coating program, anhydrous chlorobenzene (120 µL) was gently dropped on the spinning substrate. The films were then heated at 95 °C for 1 h in the glovebox.

After drying perovskite coated films completely, the HTM (70 µL) was deposited onto the perovskite layer by spin‐coating at 4000 rpm for 20 s. The HTM was prepared by dissolving 28.9 mg Spiro‐OMeTAD, 11.5 µL 4‐*tert*‐butylpyridine (tBP), 7.0 µL of a stock solution of 520 mg mL^−1^ lithium bis(trifluoromethylsulphonyl)imide (Li‐TFSI) in acetonitrile and 9.0 µL of a stock solution of 100 mg mL^−1^ FK102 Co(III) PF6 salt in acetonitrile, in 400 µL chlorobenzene. For the fabrication of SWCNT–HTM‐based device, dispersion of SWCNTs in chlorobenzene (1 mg mL^−1^) was spin‐coated onto the perovskite layer at 4000 rpm for 20 s. After the HTMs deposition, the films were stored overnight in a dry air desiccator. Finally, 50 nm gold electrodes were thermally evaporated at a rate of 1 Å s^−1^ under a high vacuum through a shadow mask.

*Characterization*: Top‐view and cross‐sectional SEM images were obtained using an Inspect F50 SEM (FEI) with accelerating voltage of 20 kV. X‐ray diffraction (XRD) of TiO_2_ NF films on FTO was carried out using a Bruker D8 diffractometer with Cu Kα source and parallel beam optics equipped with a PSD LynxEye silicon strip detector. The incident beam was kept at an angle of 1° and the angular range of diffraction patterns was collected at 10° < 2θ < 66° with a step size of 0.05° at 2 s per step. The anatase phase of TiO_2_ was confirmed by comparing the patterns from the Inorganic Crystal Structure Database (ICSD). Raman confocal spectroscopy and spectral mapping were completed using a Witec Alpha 300RS with a 40× objective and 532 nm laser excitation. Raman single spectra were acquired with integration times of 5 s and three accumulations. The Raman spectral image was obtained by collecting a series of 100 × 100 single spectra (0.2 s integration per spectrum) over an area of 20 × 20 µm. The optical transmittances of the films on glass slides were analyzed using a Varian Cary 50G UV–vis spectrophotometer at wavelengths ranging from 400 to 1000 nm. Sheet resistance measurements were performed on the same films using a four point probe technique (KeithLink Technology Co., Ltd, Taiwan). The electrochemical impedance spectroscopy was measured with an Autolab PGSTAT128N on the photoelectrodes using a half cell configuration in 0.1 m NaCl. Analysis was completed in the dark with 0 V bias with 10 mV modulation over the frequency range of 100 000–0.1 Hz.

The photocurrent–voltage (*J–V*) characteristics were analyzed using a Keithley 2400 SMU instrument and recorded using a custom LabView Virtual Instrument program. A standard silicon test cell with NIST‐traceable certification was used to calibrate the power density as 100 mW cm^−2^ at the sample plane of the collimated a 150W xenon‐arc light source (Newport), which was passed through an AM 1.5G filter. The scan rate and delay time are 200 mV s^−1^ and 30 ms, respectively. The active area of the fabricated devices was 0.14 cm^2^. The devices were masked with an aperture mask (with an area of 0.081 cm^2^) and tested in air atmosphere without encapsulation. No device preconditioning, such as prolonged light soaking or forward voltage bias, and no equilibration time was used. The IPCE spectra as a function of wavelength ranging from 300 to 800 nm were taken by passing chopped light from a xenon source through a monochromator and onto the devices. The light intensity of the illumination source was adjusted using a photodiode detector (silicon calibrated detector, Newport).

*Computational Detail*: All DFT computations were performed with the Vienna ab initio simulation package (VASP, version 5.4.1) using the projector‐augmented wave (PAW) method.^69^, ^70^ Electron–ion interactions were described using standard PAW potentials with valence configurations of 3s^2^3p^6^4s^2^3d^2^ for Ti (Ti_sv_GW), 2s^2^2p^2^ for C (C_GW_new), 2s^2^2p^4^ for O (O_GW_new), and 1s^1^ for H (H_GW). A plane‐wave basis set was employed to expand the smooth part of wave functions with a cutoff kinetic energy of 520 eV. The electron–electron exchange and correlation interactions were parameterized by Perdew–Burke–Ernzerhhof (PBE),^71^ a form of the general gradient approximation (GGA), was used throughout.

To simulate the interaction between the SWCNT and anatase TiO_2_, the anatase TiO_2_ (101) surface was employed since it is the most stable.^72^ For structural relaxations of the anatase (101) surfaces, a 12‐layer slab for the (2 × 2) surface cell with 144 atoms was enclosed in a supercell with sufficiently large vacuum regions of 15 Å to ensure the periodic images to be well separated. During the geometry optimizations, all atoms were allowed to relax until the Hellmann–Feynman forces were smaller than 0.001 eV Å^–1^. The convergence criterion for the electronic self‐consistent loop was set to 10^−5^ eV. Brillouin‐zone integrations were performed using a gamma‐centered (1 × 3 × 1) *k*‐point grid. The corresponding *k*‐mesh densities and the cutoff kinetic energy have been justified in our previous studies.^73^, ^74^ Since P3‐SWCNT used in this study has 3%–6% carboxylic acid groups, the adsorption of SWCNT was investigated through the interaction between carboxylic acid group with the Ti and O atoms in the (101) surface. The detailed atomic structure at the interface of SWCNT and TiO_2_ can be found in Figure 6b. The energy level versus the SHE is calculated according to the Equation

$$U\left(\mathrm{vs}\;\mathrm{SHE},V\right)=(E_{\mathrm{vac}}-E_{b}-E_{\mathrm{SHE}})/e$$

where *E*_vac_, *E*_b_, and *E*_SHE_ are vacuum energy, the energy of electronic bands, and the absolute energy of SHE (which is 4.44 eV), respectively. And “*e*” represents the electron charge here.

## Supporting information

As a service to our authors and readers, this journal provides supporting information supplied by the authors. Such materials are peer reviewed and may be re‐organized for online delivery, but are not copy‐edited or typeset. Technical support issues arising from supporting information (other than missing files) should be addressed to the authors.

Supplementary
