# Supplementary material for: Carbon Nanotubes in TiO2 Nanofiber Photoelectrodes for High‐Performance Perovskite Solar Cells
Source: Adv Sci (Weinh). 2017 Jan 20;4(4):1600504. doi: 10.1002/advs.201600504 (PMC5396161; doi:10.1002/advs.201600504)
Supplement: Supplementary file 1 — Supplementary [file ADVS-4-na-s001.pdf]

Copyright WILEY-VCH Verlag GmbH & Co. KGaA, 69469 Weinheim, Germany, 2017.

ADVANCED SCIENCE
Open Access

# Supporting Information

for *Adv. Sci.,* DOI: 10.1002/advs.201600504

# Carbon Nanotubes in $TiO_2$ Nanofiber Photoelectrodes for High-Performance Perovskite Solar Cells

*Munkhbayar Batmunkh, Thomas J. Macdonald, Cameron J. Shearer, Munkhjargal Bat-Erdene, Yun Wang, Mark J. Biggs, Ivan P. Parkin, Thomas Nann, and Joseph G. Shapter**

((Supporting Information can be included here using this template))

Copyright WILEY-VCH Verlag GmbH & Co. KGaA, 69469 Weinheim, Germany, 2013.

# Supporting Information

## Carbon Nanotubes in $TiO_2$ Nanofiber Photoelectrodes for High Performance Perovskite Solar Cells

*Munkhbayar Batmunkh, Thomas J. Macdonald, Cameron J. Shearer, Munkhjargal Bat-Erdene, Yun Wang, Mark J. Biggs, Ivan P. Parkin, Thomas Nann, and Joseph G. Shapter **

Munkhbayar Batmunkh, Prof. Mark J. Biggs
School of Chemical Engineering,
The University of Adelaide,
Adelaide, South Australia 5005, Australia

Munkhbayar Batmunkh, Dr. Cameron J. Shearer, Munkhjargal Bat-Erdene, Prof. Joseph G. Shapter
School of Chemical and Physical Sciences,
Flinders University,
Bedford Park, Adelaide, South Australia 5042, Australia
E-mail: joe.shapter@flinders.edu.au

Dr. Thomas J. Macdonald, Prof. Ivan P. Parkin
Department of Chemistry,
University College London,
London, UK

Dr. Yun Wang
Centre for Clean Environment and Energy,
Griffith School of Environment, Gold Coast Campus, Griffith University,
Queensland 4222, Australia

Prof. Mark J. Biggs
School of Science,
Loughborough University,
Loughborough, Leicestershire, LE11 3TU, UK

Prof. Thomas Nann
MacDiarmid Institute for Advanced Materials and Nanotechnology,
School of Chemical and Physical Sciences, Victoria University of Wellington,
Wellington, New Zealand

**Keywords:** Photovoltaic, perovskite solar cells, photoelectrodes, $TiO_2$ nanofibers, carbon nanotubes

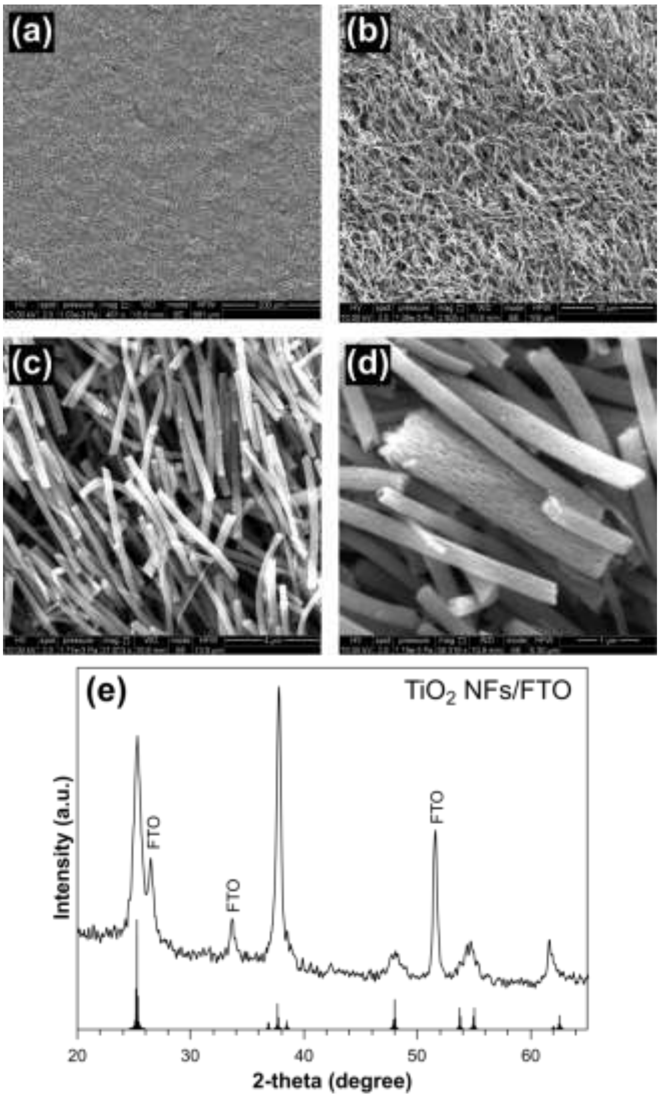


**Figure S1.** (a-d) SEM images of the electrospun $TiO_2$ NFs. (e) XRD pattern of $TiO_2$ NFs on FTO confirming the anatase phase with reference to 9853-ICSD.

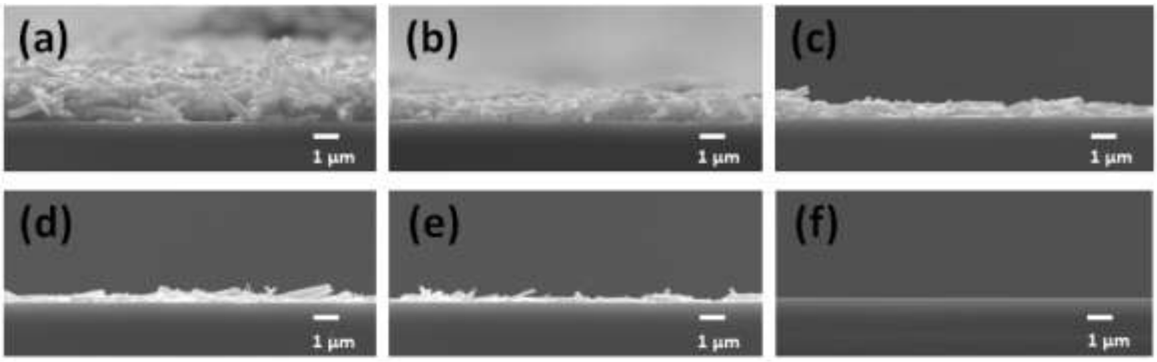


**Figure S2.** Cross sectional SEM images of $TiO_2$ NF photoelectrodes with the thickness of (a) ~2200 nm, (b) ~1300 nm, (c) ~580 nm, (d) ~400 nm, (e) ~285 nm and (f) 0 nm (planar).

**Table S1.** PV parameters of best performing PSCs fabricated with different $TiO_2$ NF thicknesses based photoelectrodes (extracted from the *J–V* characteristics reported in Figure 2a). The thicknesses of $TiO_2$ NF films for the fabrication of PSC devices are illustrated in Figure S2. The average PCEs of the cells were calculated based on at least five identical devices.

| $TiO_2$ NFs thickness | $J_{sc}$ (mA cm$^{-2}$) | $V_{oc}$ (V) | *FF* | PCE (%) | Average PCE (%) |
|---|---|---|---|---|---|
| 2200 nm | 11.58 | 0.75 | 0.58 | 5.01 | 4.77 ± 0.27 |
| 1300 nm | 13.19 | 0.77 | 0.61 | 6.13 | 5.50 ± 0.47 |
| 580 nm | 15.55 | 0.84 | 0.58 | 7.54 | 7.21 ± 0.36 |
| 400 nm | 15.91 | 0.87 | 0.62 | 8.56 | 8.21 ± 0.46 |
| 285 nm | 14.86 | 0.87 | 0.62 | 8.07 | 7.74 ± 0.44 |
| 0 nm (planar) | 12.87 | 0.94 | 0.58 | 7.02 | 6.70 ± 0.44 |

**Table S2.** PV parameters of best performing PSC devices fabricated with different types of CNTs incorporated $TiO_2$ NF photoelectrodes (extracted from the *J–V* characteristics reported in Figure 2b). ~400 nm $TiO_2$ NF films were chosen for these cells. The concentration of CNTs in the $TiO_2$ NF-CNT hybrid was 0.02 wt%. The average PCEs of the cells were calculated based on at least five identical devices.

| Device | $J_{sc}$ (mA cm$^{-2}$) | $V_{oc}$ (V) | *FF* | PCE (%) | Average PCE (%) |
|---|---|---|---|---|---|
| $TiO_2$ NFs-only | 15.91 | 0.87 | 0.62 | 8.56 | 8.21 ± 0.46 |
| $TiO_2$ NFs-DWCNTs | 16.71 | 0.88 | 0.62 | 9.04 | 8.81 ± 0.20 |
| $TiO_2$ NFs-MWCNTs | 17.07 | 0.86 | 0.62 | 9.08 | 8.97 ± 0.15 |
| $TiO_2$ NFs-SWCNTs | 17.20 | 0.93 | 0.62 | 9.91 | 9.69 ± 0.23 |

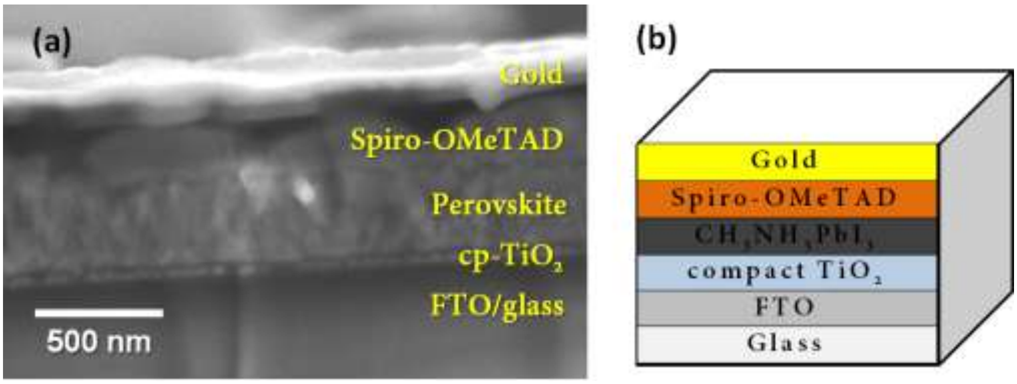


**Figure S3.** (a) Cross sectional SEM image and (b) schematic illustration of the planar PSC device structure.

The measurement of sheet resistance ($R_s$) of the $TiO_2$ NF-SWCNT thin films (on glass substrate) was carried out using a four point probe to investigate the mechanism of enhancement in the $J_{sc}$ value of the devices. As shown in Figure S4, the $R_s$ of $TiO_2$ NF films decreased gradually with increasing concentration of SWCNTs. This decrease in the $R_s$ of the films is attributed to the high conductivity of SWCNTs that decreases the interfacial resistance between $TiO_2$ NFs.

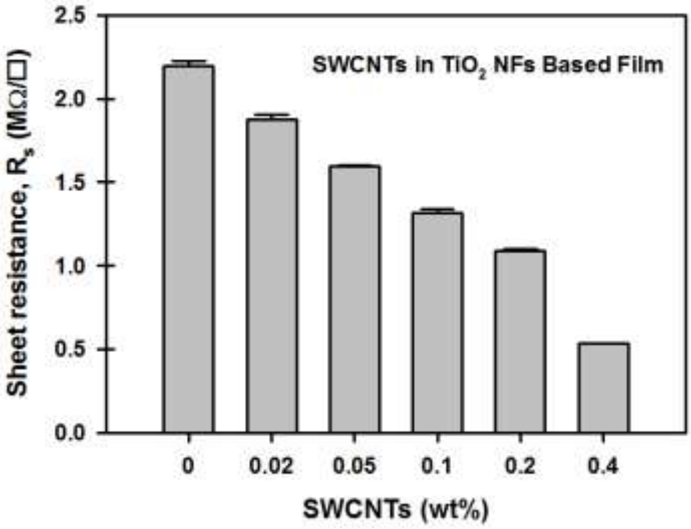


**Figure S4.** $R_s$ of the $TiO_2$ NF films with various SWCNT loadings. Error bars are calculated from five different measurements.

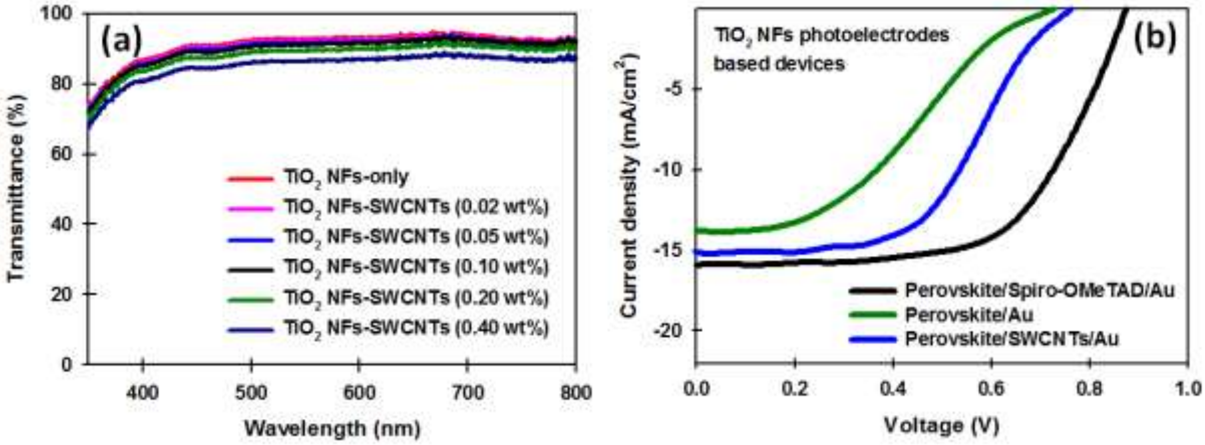


**Figure S5.** (a) Optical transmittance of the $TiO_2$ NF based films with different concentrations of SWCNTs. (b) *J–V* curves of $TiO_2$ NF-only photoelectrodes based PSCs with different hole transporting materials (HTMs).

**Table S3.** PV parameters of PSCs with and without SWCNTs in the photoelectrodes measured at forward and reverse *J–V* scans.

| Device | Scan direction | $J_{sc}$ (mA cm$^{-2}$) | $V_{oc}$ (V) | *FF* | PCE (%) |
|---|---|---|---|---|---|
| $TiO_2$ NFs-only | Forward | 14.96 | 0.87 | 0.52 | 6.78 |
| | Reverse | 15.38 | 0.87 | 0.61 | 8.16 |
| $TiO_2$ NFs-SWCNTs | Forward | 18.76 | 0.92 | 0.57 | 9.86 |
| | Reverse | 18.89 | 0.93 | 0.64 | 11.44 |

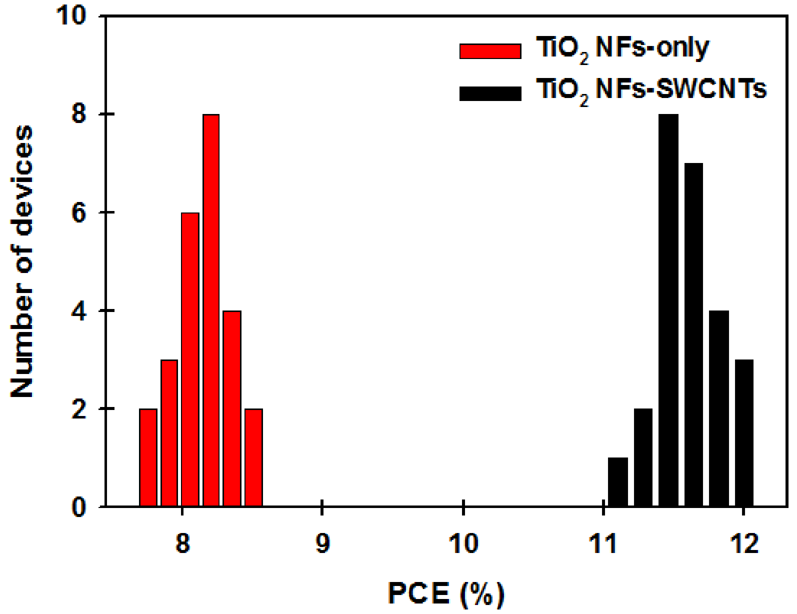


**Figure S6.** Histograms of PCE for the $TiO_2$ NFs-only and $TiO_2$ NF-SWCNT PSCs (measurement of 25 cells for each device structure).

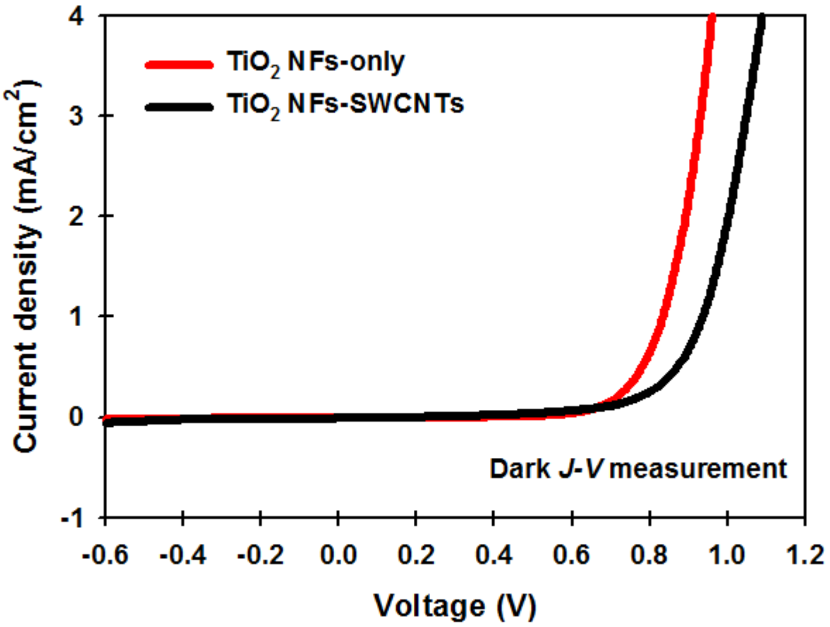


**Figure S7**. Dark *J-V* curves of PSCs fabricated based on $TiO_2$ NFs-only and $TiO_2$ NFs-SWCNTs photoelectrodes.

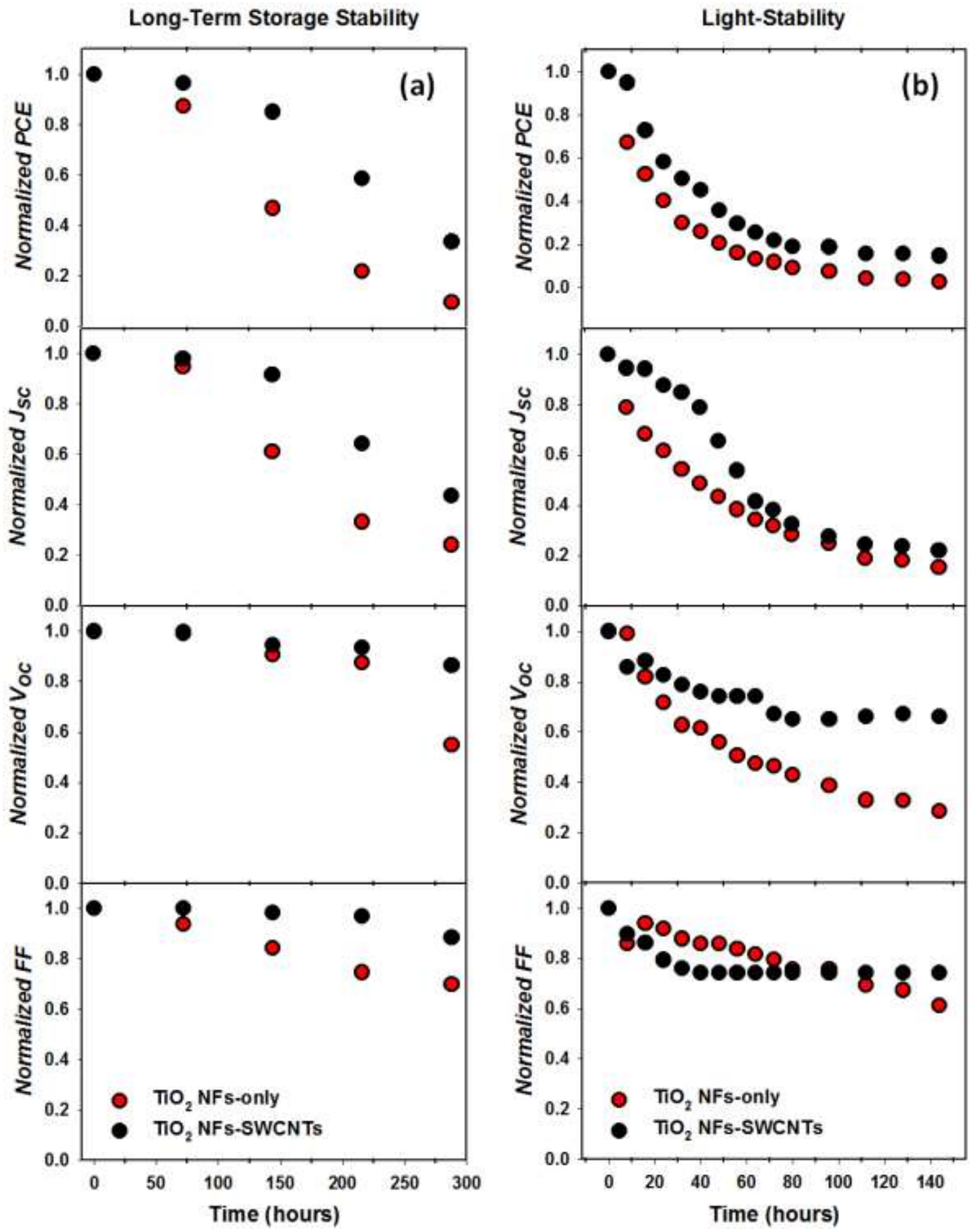


**Figure S8.** (a) Long-term storage- and (b) light-stability of the PSCs fabricated with and without SWCNTs in the $TiO_2$ NF photoelectrodes. For the long-term storage-stability, the fabricated cells were kept in the dark in ambient conditions for 288 h. The devices were not encapsulated for the stability test. For the light-stability test, the devices were exposed to continuous light illumination (100 mW $cm^{-2}$) in ambient conditions and the data were obtained in reverse scan direction at every 8 min. In Y-axis (normalized PV parameters), PCE(in), $J_{sc}$(in), $V_{oc}$(in) and $FF$(in) represents the initial (0 hr) PV values of the devices.

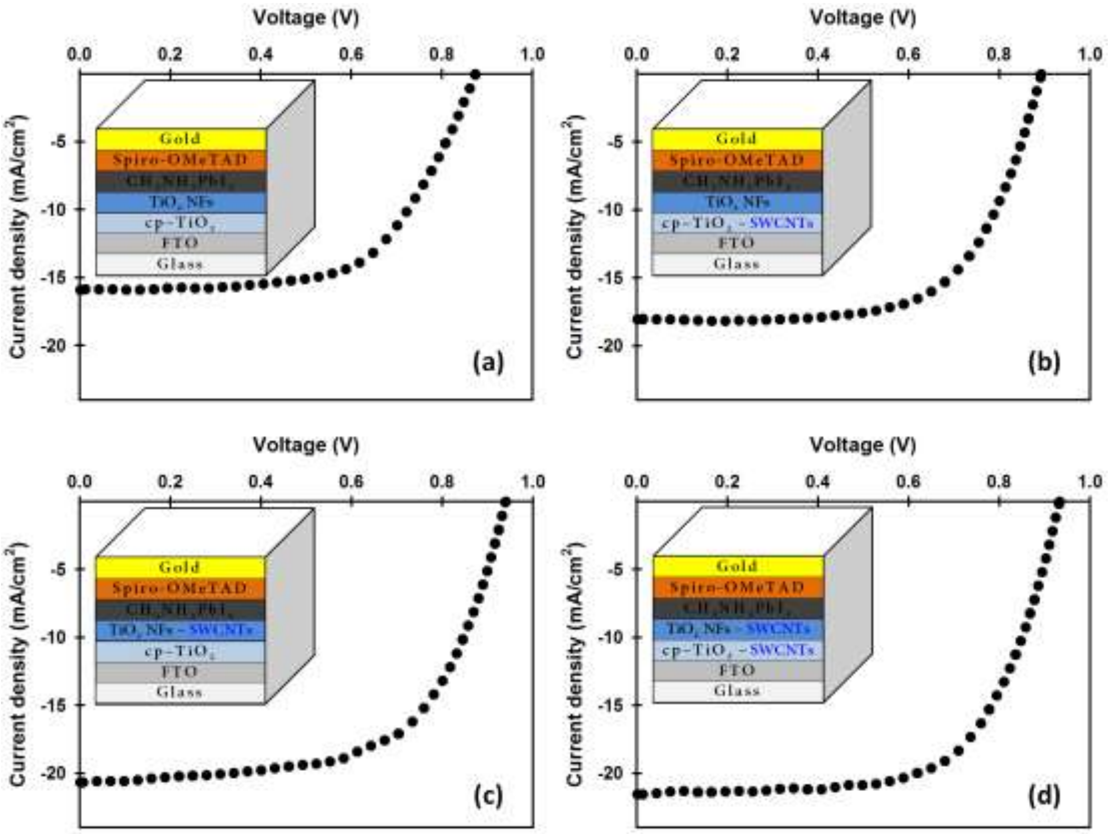


**Figure S9**. *J–V* curves of PSCs based on (a) compact (*cp*)-$TiO_2$ and $TiO_2$ NFs-only photoeletrode without any SWCNTs, (b) SWCNTs incorporated *cp*-$TiO_2$ layer and $TiO_2$ NFs-only photoelectrode, (c) *cp*-$TiO_2$ layer and SWCNTs incorporated $TiO_2$ NFs photoelectrode, and (d) SWCNTs incorporated into both *cp*-$TiO_2$ and $TiO_2$ NFs photoelectrode. An aperture mask was used during the *J–V* test.

**Table S4.** Detailed PV parameters of PSCs based on (Structure A) *cp*-$TiO_2$ and $TiO_2$ NFs-only photoeletrode without any SWCNTs, (Structure B) SWCNTs incorporated *cp*-$TiO_2$ layer and $TiO_2$ NFs-only photoelectrode, (Structure C) *cp*-$TiO_2$ layer and SWCNTs incorporated $TiO_2$ NFs photoelectrode, and (Structure D) SWCNTs incorporated into both *cp*-$TiO_2$ and $TiO_2$ NFs photoelectrode. The device structures are shown in the inset of Figure S9.

| Device | $J_{sc}$ (mA cm$^{-2}$) | $V_{oc}$ (V) | *FF* | PCE (%) | Average PCE (%) |
|---|---|---|---|---|---|
| Structure A | 15.91 | 0.87 | 0.62 | 8.56 | 8.21 ± 0.46 |
| Structure B | 18.02 | 0.89 | 0.64 | 10.38 | 9.88 ± 0.43 |
| Structure C | 20.68 | 0.94 | 0.62 | 12.03 | 11.51 ± 0.40 |
| Structure D | 21.51 | 0.93 | 0.65 | 13.04 | 12.75 ± 0.43 |

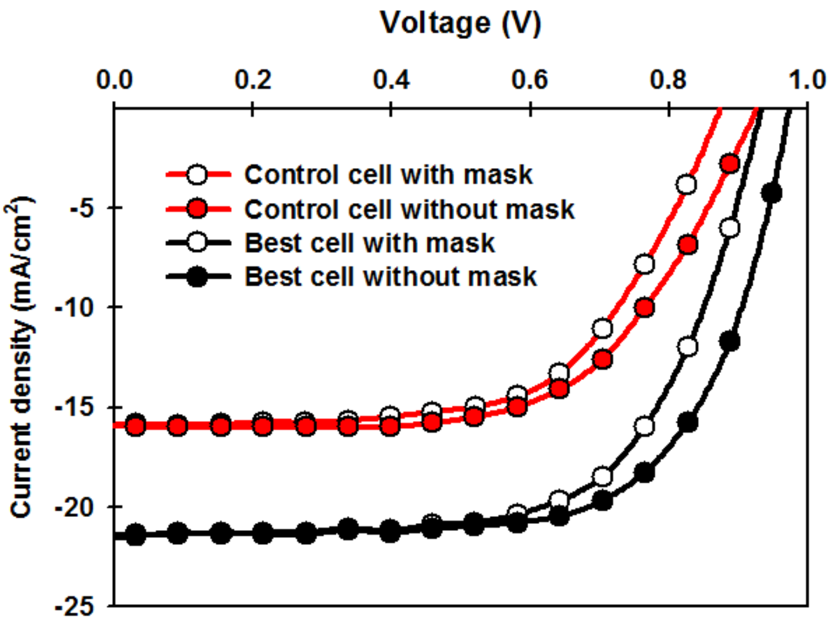


**Figure S10.** Effect of aperture masking on the *J–V* measurement of the PSC devices. The control cell is fabricated with the device structure shown in Figure 1a (without any SWCNT), while the best cell is made of structure such as that illustrated in the inset of Figure 9. The overlapped area of FTO electrode (anode) and gold electrode (cathode) was 0.14 $cm^2$. The aperture mask with an area of 0.081 $cm^2$ was used for the measurement with mask.
